# Supplementary figures and images for: Transcriptomic analysis of cellular senescence induced by ectopic expression of ATF6α in human breast cancer cells
Source: PLoS One. 2024 Oct 28;19(10):e0309749. doi: 10.1371/journal.pone.0309749 (PMC11515977; doi:10.1371/journal.pone.0309749)

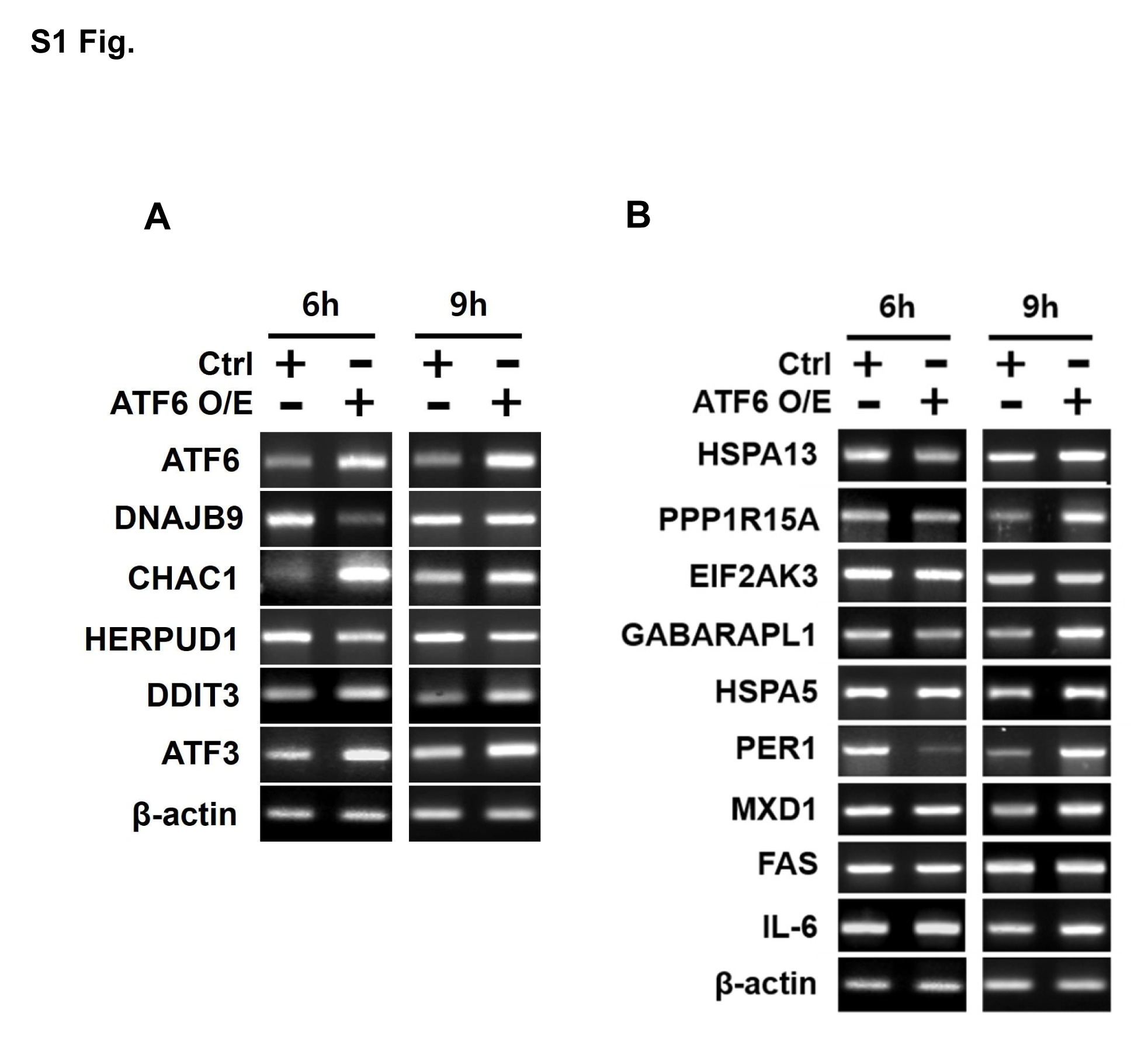

Supplement: S1 Fig — (A, B) MCF-7 cells were transfected with ATF6α cDNA or an empty vector DNA as a control. RT-PCR was conducted to assess the mRNA levels of the indicated genes selected from the 6h-DEGs (A) or 9h-DEGs (B) in MCF-7 cells after ATF6α ectopic expression. Data represent three independent experiments with similar results. Ctrl, control; ATF6 O/E, ATF6α overexpression. (TIF) [file pone.0309749.s001.tif]

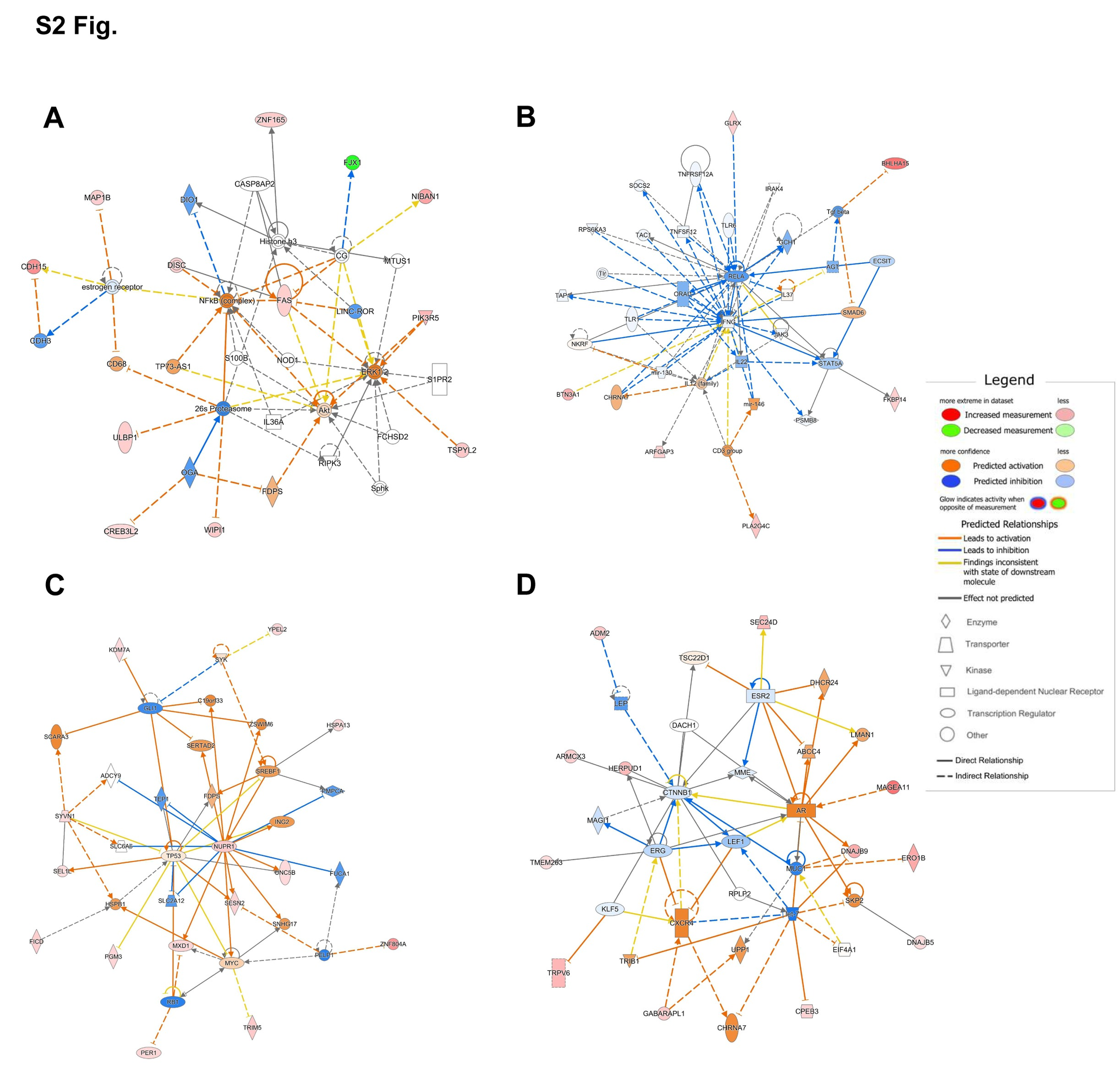

Supplement: S2 Fig — (A) Gene network of 9h-DEGs with a score of 24, associated with cell cycle, cellular development, and connective tissue development and function. (B) Gene network of 9h-DEGs with a score of 20, linked to cellular development, cellular growth and proliferation, and cellular movement. (C) Gene network of 9h-DEGs with a score of 18, related to hematological system development and function, humoral immune response, and lymphoid tissue structure and development. (D) Gene network of 9h-DEGs with a score of 8, associated with cell-to-cell signaling and interaction, hematological system development and function, and immune cell trafficking. The legend illustrates the relationship between molecules within the network and their activation state. (TIF) [file pone.0309749.s002.tif]

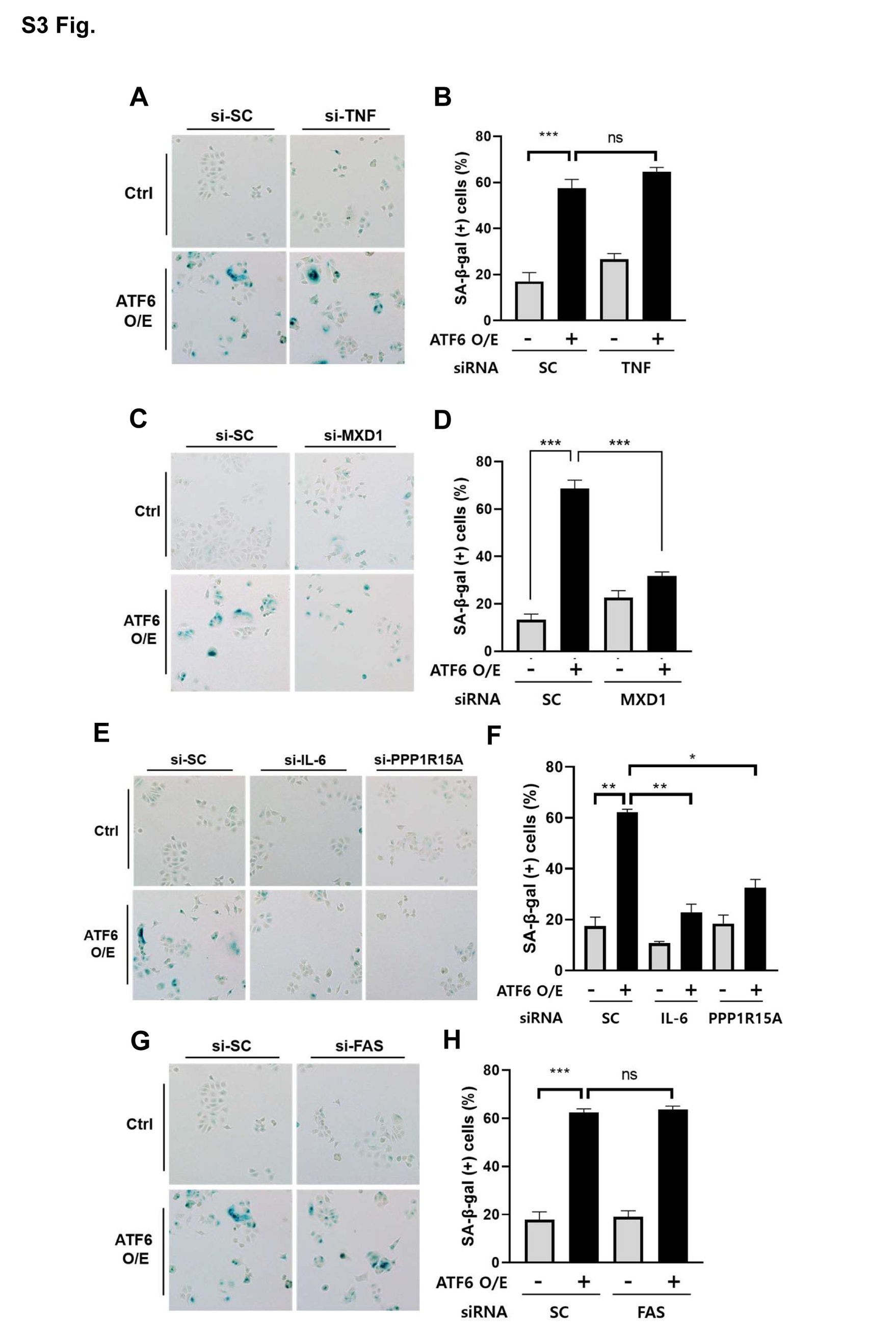

Supplement: S3 Fig — MCF-7 cells were co-transfected with ATF6α cDNA, and control siRNA (SC) or siRNA for the indicated genes. After 5 days, SA-β-gal staining was performed. (A, C, E, G) Light microscopic images represent three independent experiments. (B, D, F, H) Percentages of SA-β-gal (+) cells were plotted. (I, K, M) Light microscopic images represent three independent experiments. (J, L, N) Percentages of SA-β-gal (+) cells were plotted. Data represent the mean of triplicate determinations ± S.D. (O, P) MCF-7 cells were transfected with ATF6α cDNA or an empty vector and treated with 100 nM Ravoxertinib, an ERK inhibitor, or 500 nM Trametinib, a MEK1/2 inhibitor. After 5 days, SA-β-gal staining was performed. Data represent the mean of triplicate determinations ± S.D. Ctrl, control; ATF6 O/E, ATF6α overexpression. * P<0.05; ** P<0.01; *** P<0.001; ns, non-significant. (ZIP) [file pone.0309749.s003.zip › S3A-S3H Fig.tif]

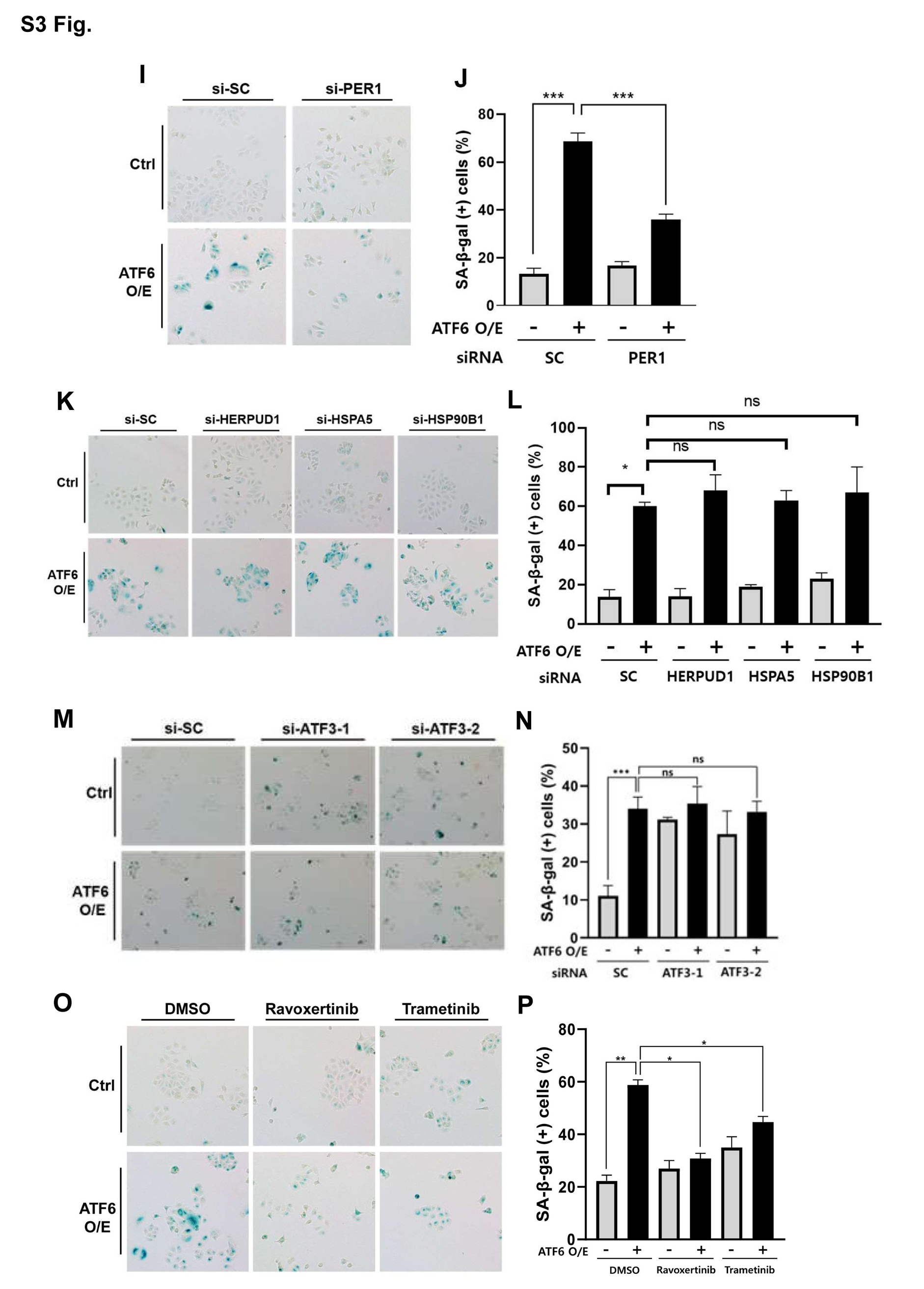

Supplement: S3 Fig — MCF-7 cells were co-transfected with ATF6α cDNA, and control siRNA (SC) or siRNA for the indicated genes. After 5 days, SA-β-gal staining was performed. (A, C, E, G) Light microscopic images represent three independent experiments. (B, D, F, H) Percentages of SA-β-gal (+) cells were plotted. (I, K, M) Light microscopic images represent three independent experiments. (J, L, N) Percentages of SA-β-gal (+) cells were plotted. Data represent the mean of triplicate determinations ± S.D. (O, P) MCF-7 cells were transfected with ATF6α cDNA or an empty vector and treated with 100 nM Ravoxertinib, an ERK inhibitor, or 500 nM Trametinib, a MEK1/2 inhibitor. After 5 days, SA-β-gal staining was performed. Data represent the mean of triplicate determinations ± S.D. Ctrl, control; ATF6 O/E, ATF6α overexpression. * P<0.05; ** P<0.01; *** P<0.001; ns, non-significant. (ZIP) [file pone.0309749.s003.zip › S3I-S3P Fig.tif]

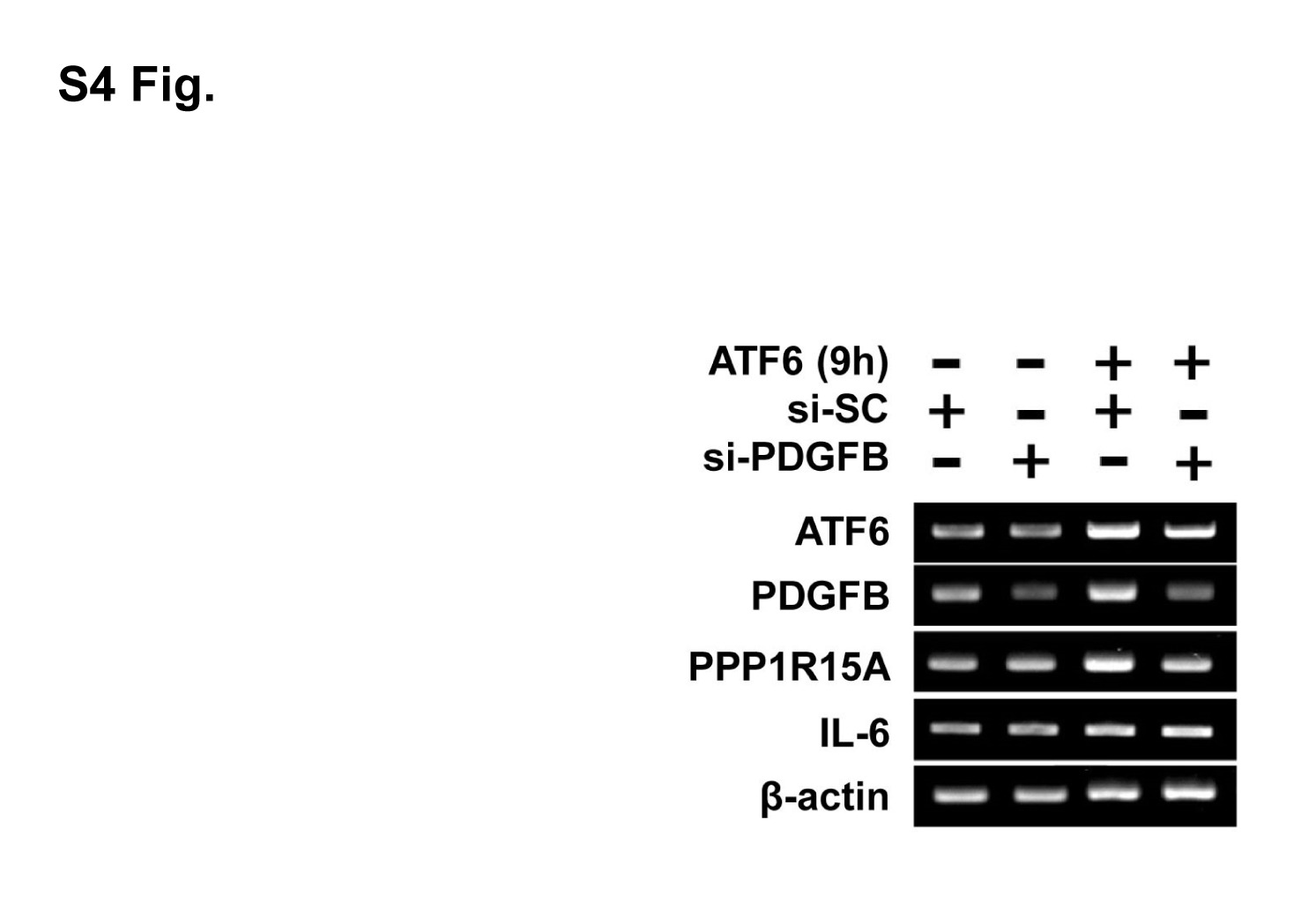

Supplement: S4 Fig — MCF-7 cells were co-transfected with ATF6α cDNA, and control siRNA (si-SC) or PDGFB siRNA. RT-PCR was conducted to assess mRNA levels of PPP1R15A and IL-6 at 9 hours after ATF6α ectopic expression. Data are representative of three independent experiments with similar results. (TIF) [file pone.0309749.s004.tif]

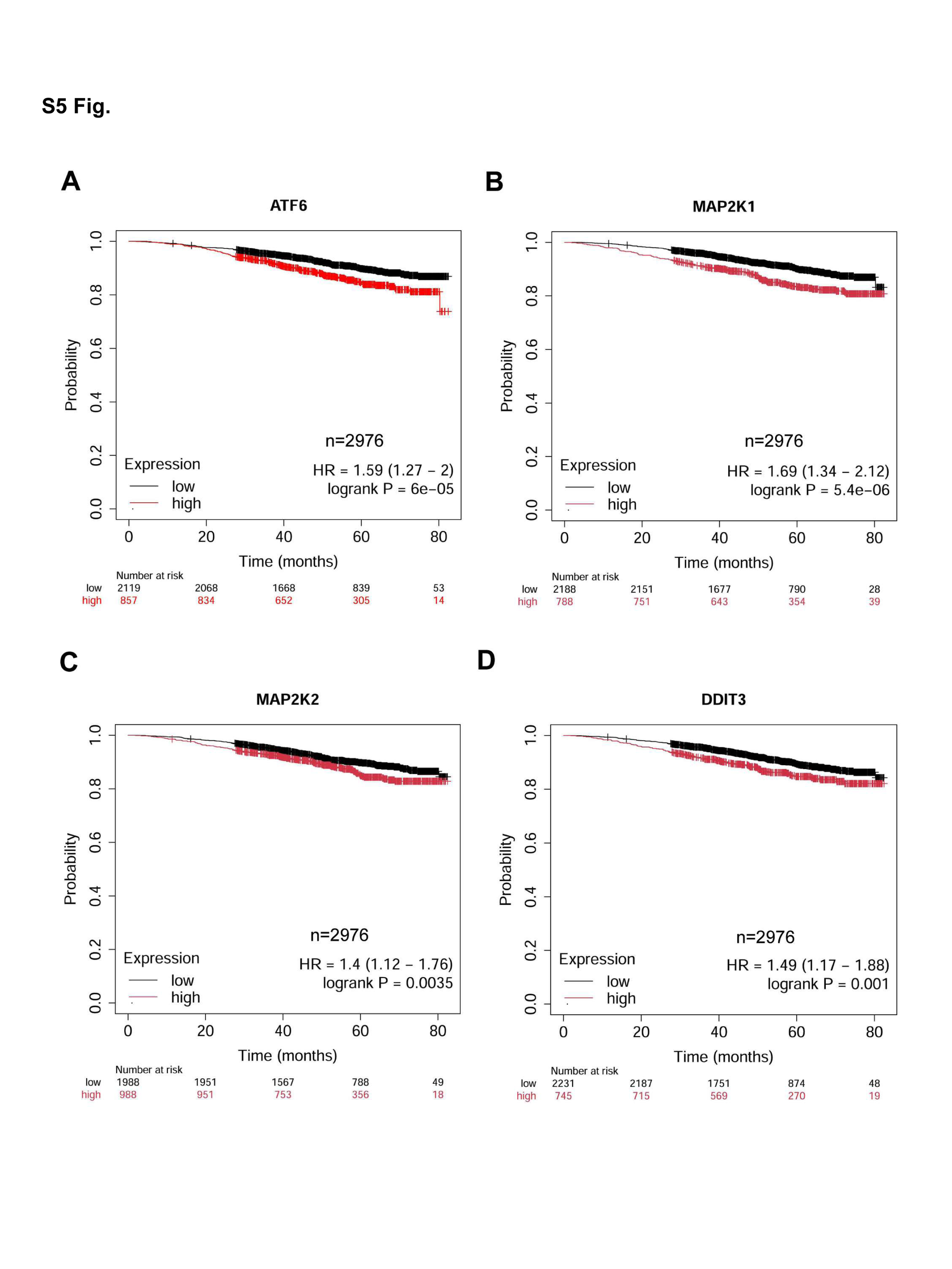

Supplement: S5 Fig — The OS of breast cancer patients with high and low expressions of ATF6α was analyzed by Kaplan-Meier plotter (https://kmplot.com/analysis). (A) The OS curves of breast cancer patients with high and low expression levels of ATF6. (B) The OS curves of breast cancer patients with high and low expression levels of MAP2K1. (C) The OS curves of breast cancer patients with high and low expression levels of MAP2K2. (D) The OS curves of breast cancer patients with high and low expression levels of DDIT3. Hazard ratio (HR), log rank P values, and the number of patients (n) were indicated in the plots. (TIF) [file pone.0309749.s005.tif]

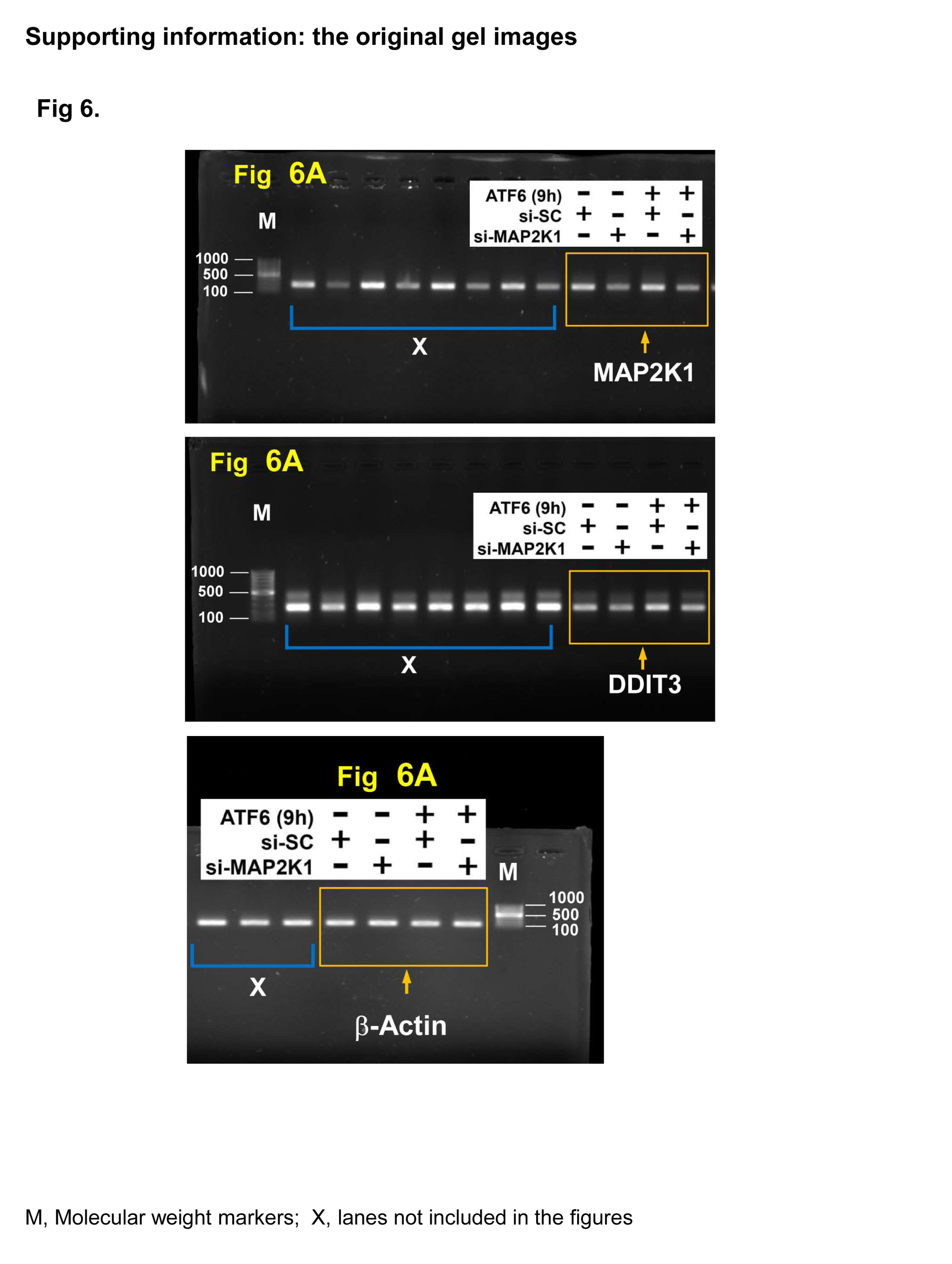

Supplement: S6 Fig — Data represnet the raw uncropped images of Fig 6A. M, Molecular weight markers; X, lanes not included in the figures. (TIF) [file pone.0309749.s006.tif]

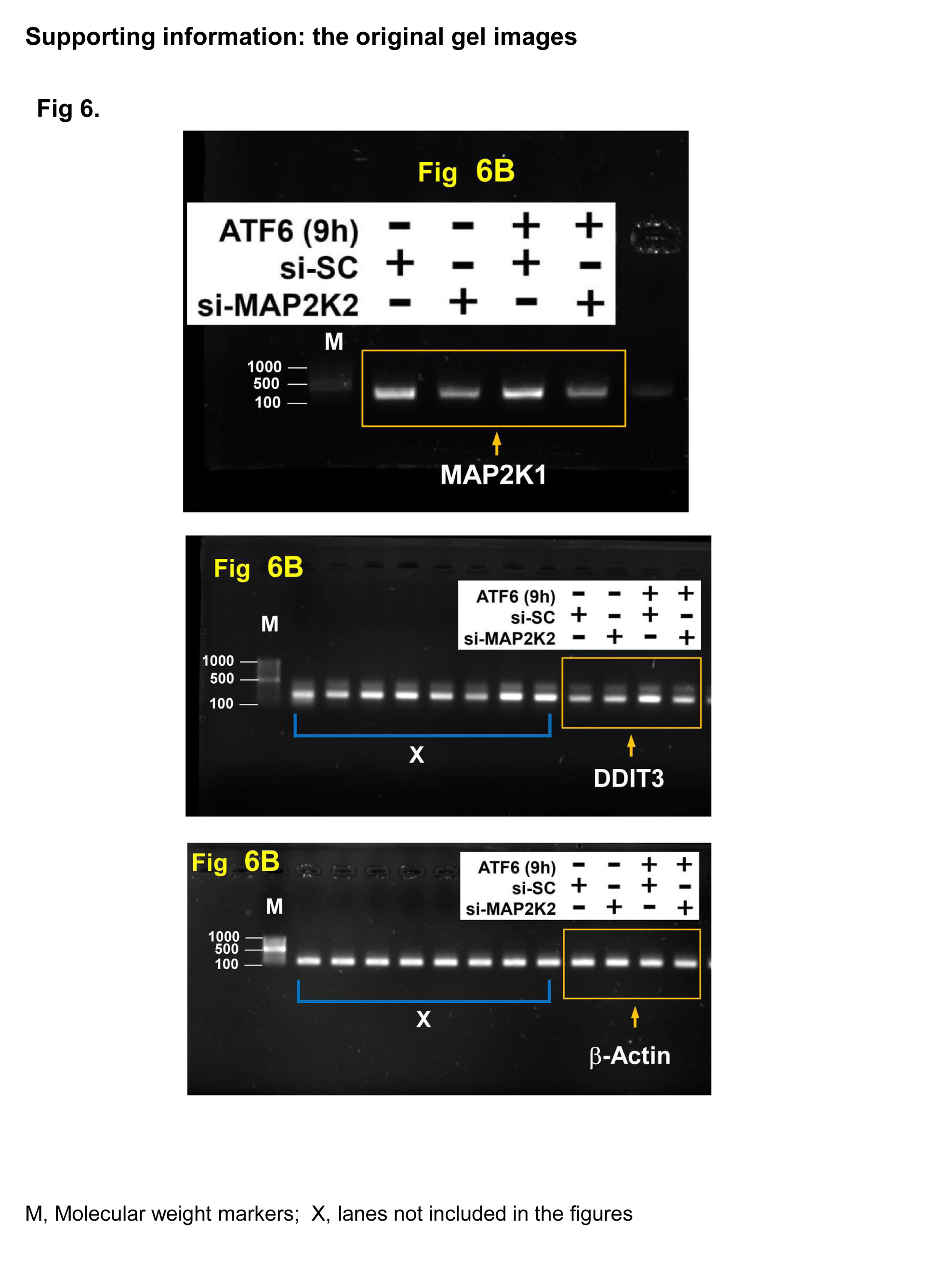

Supplement: S7 Fig — Data represnet the raw uncropped images of Fig 6B. M, Molecular weight markers; X, lanes not included in the figures. (TIF) [file pone.0309749.s007.tif]

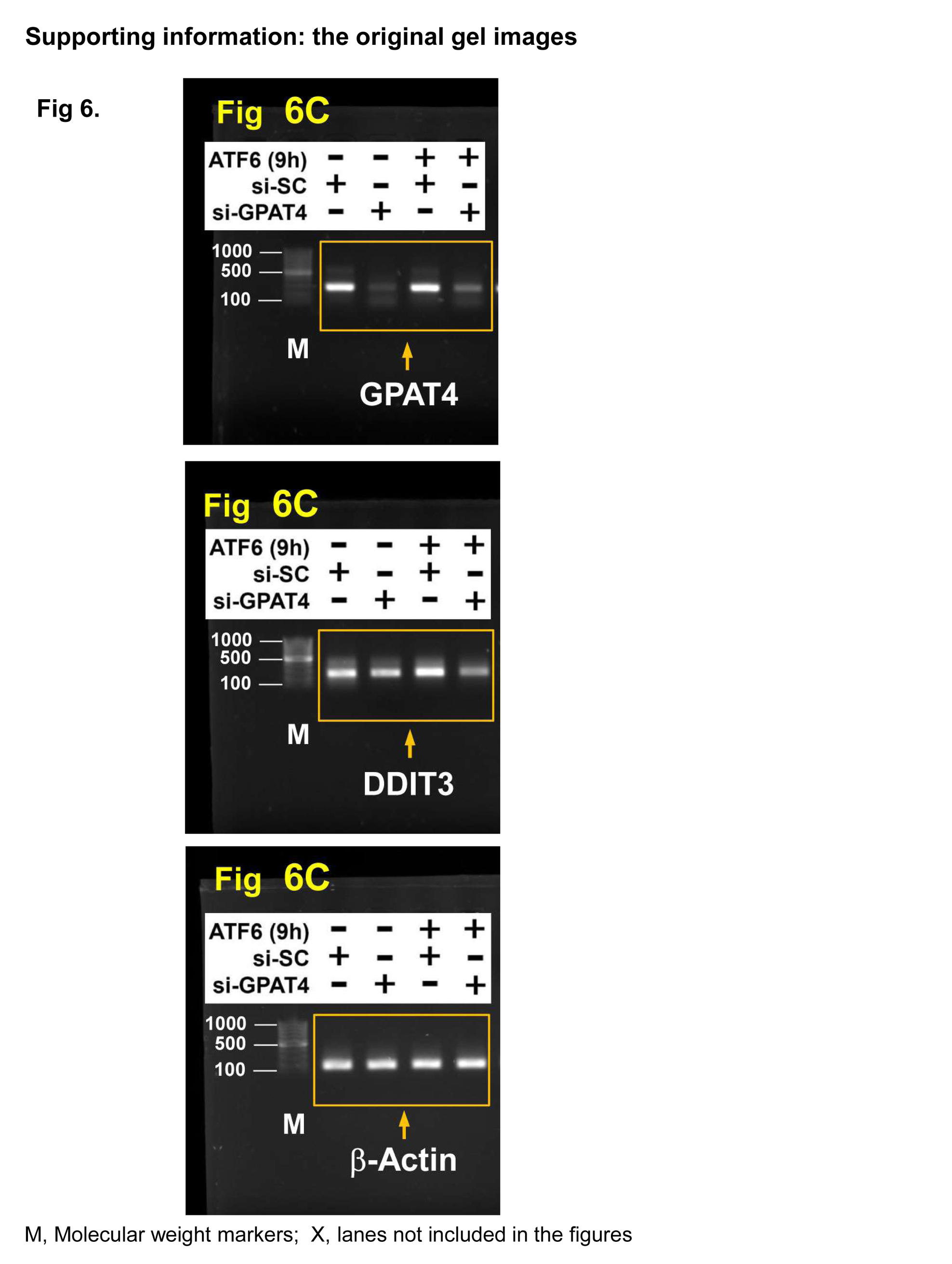

Supplement: S8 Fig — Data represnet the raw uncropped images of Fig 6C. M, Molecular weight markers; X, lanes not included in the figures. (TIF) [file pone.0309749.s008.tif]

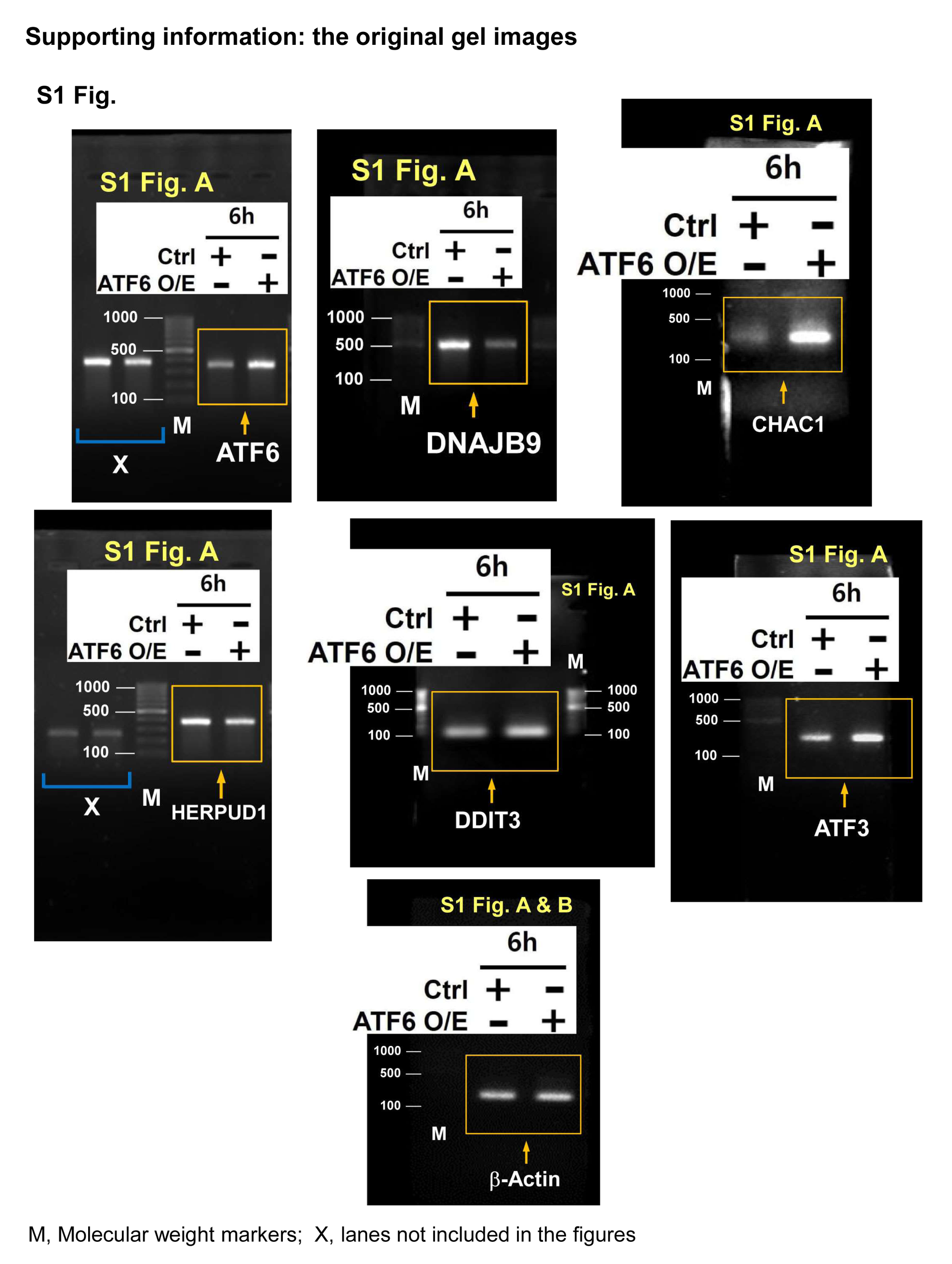

Supplement: S9 Fig — Data represnet the raw uncropped images of the 6h lane of S1A Fig. M, Molecular weight markers; X, lanes not included in the figures. (TIF) [file pone.0309749.s009.tif]

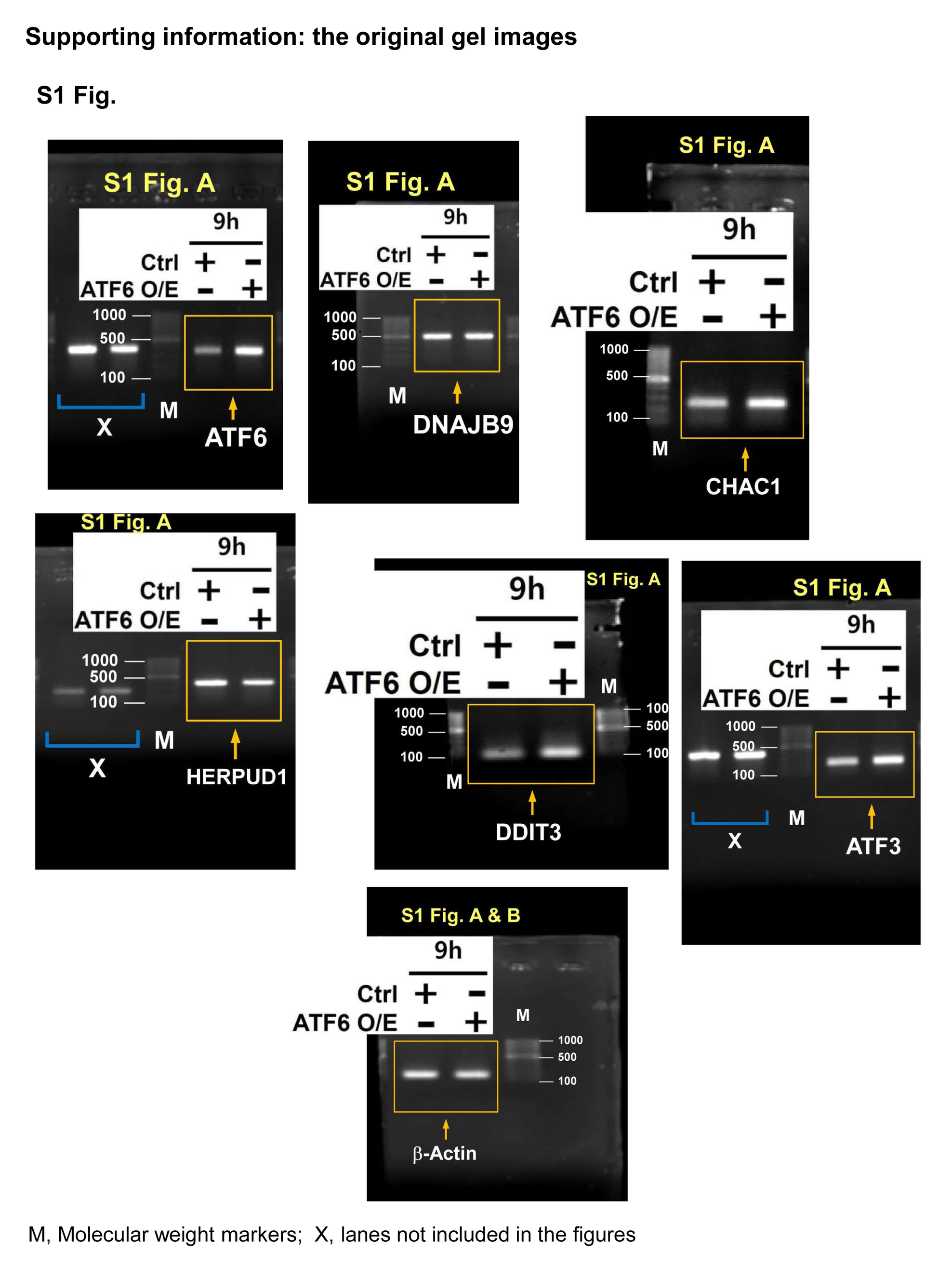

Supplement: S10 Fig — Data represnet the raw uncropped images of the 9h lane of S1A Fig. M, Molecular weight markers; X, lanes not included in the figures. (TIF) [file pone.0309749.s010.tif]

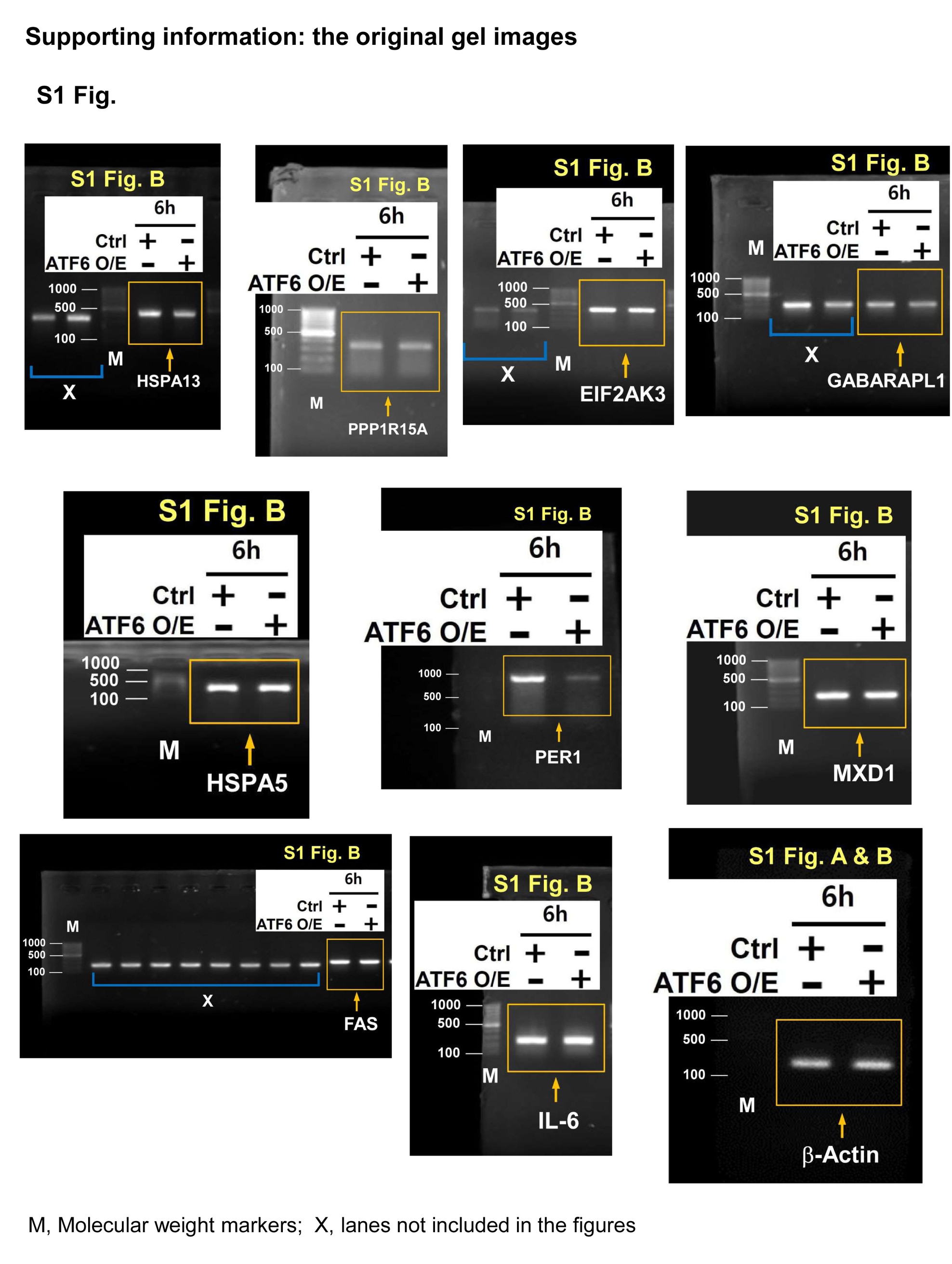

Supplement: S11 Fig — Data represnet the raw uncropped images of the 6h lane of S1B Fig. M, Molecular weight markers; X, lanes not included in the figures. (TIF) [file pone.0309749.s011.tif]

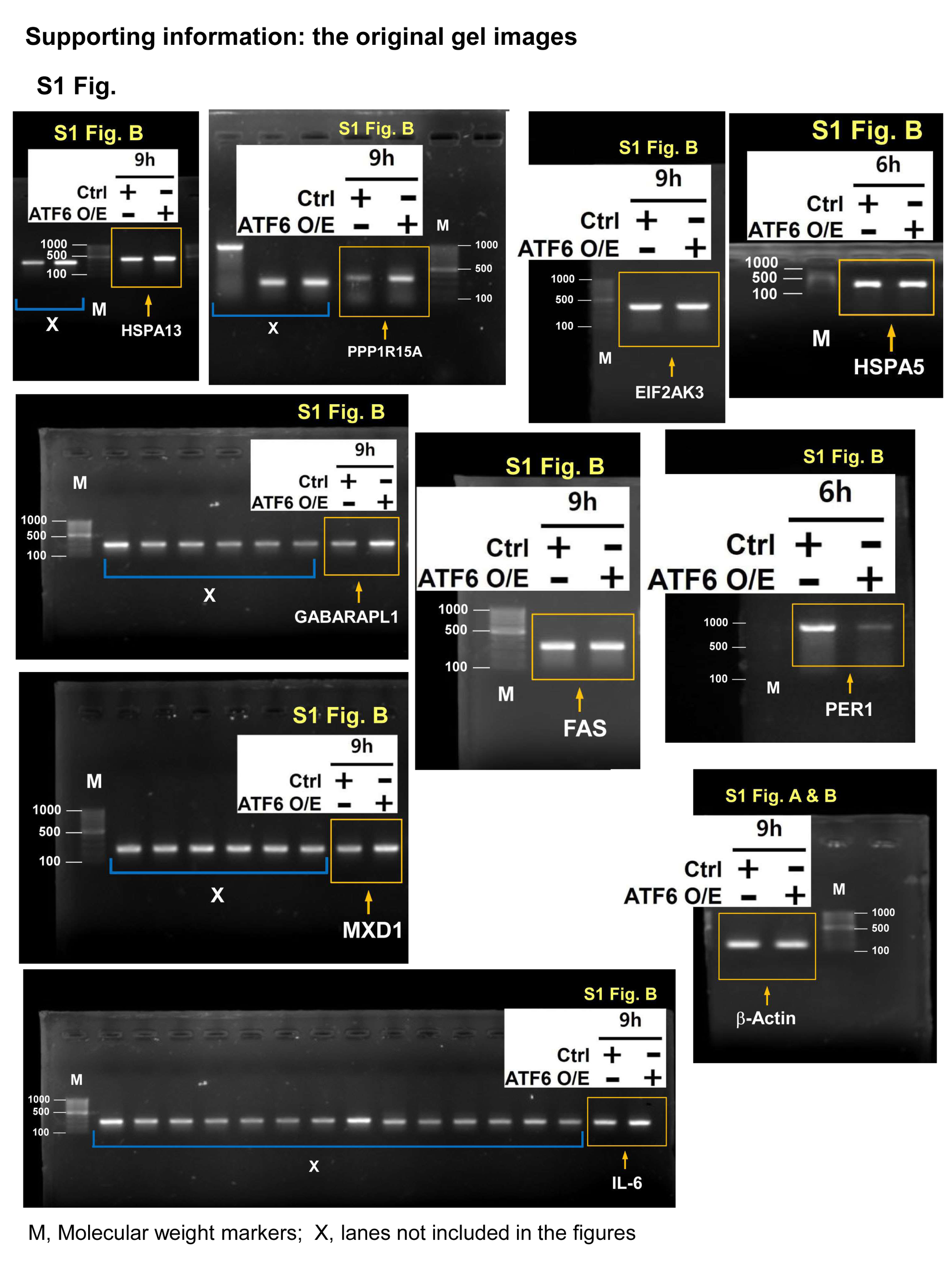

Supplement: S12 Fig — Data represnet the raw uncropped images of the 9h lane of S1B Fig. M, Molecular weight markers; X, lanes not included in the figures. (TIF) [file pone.0309749.s012.tif]

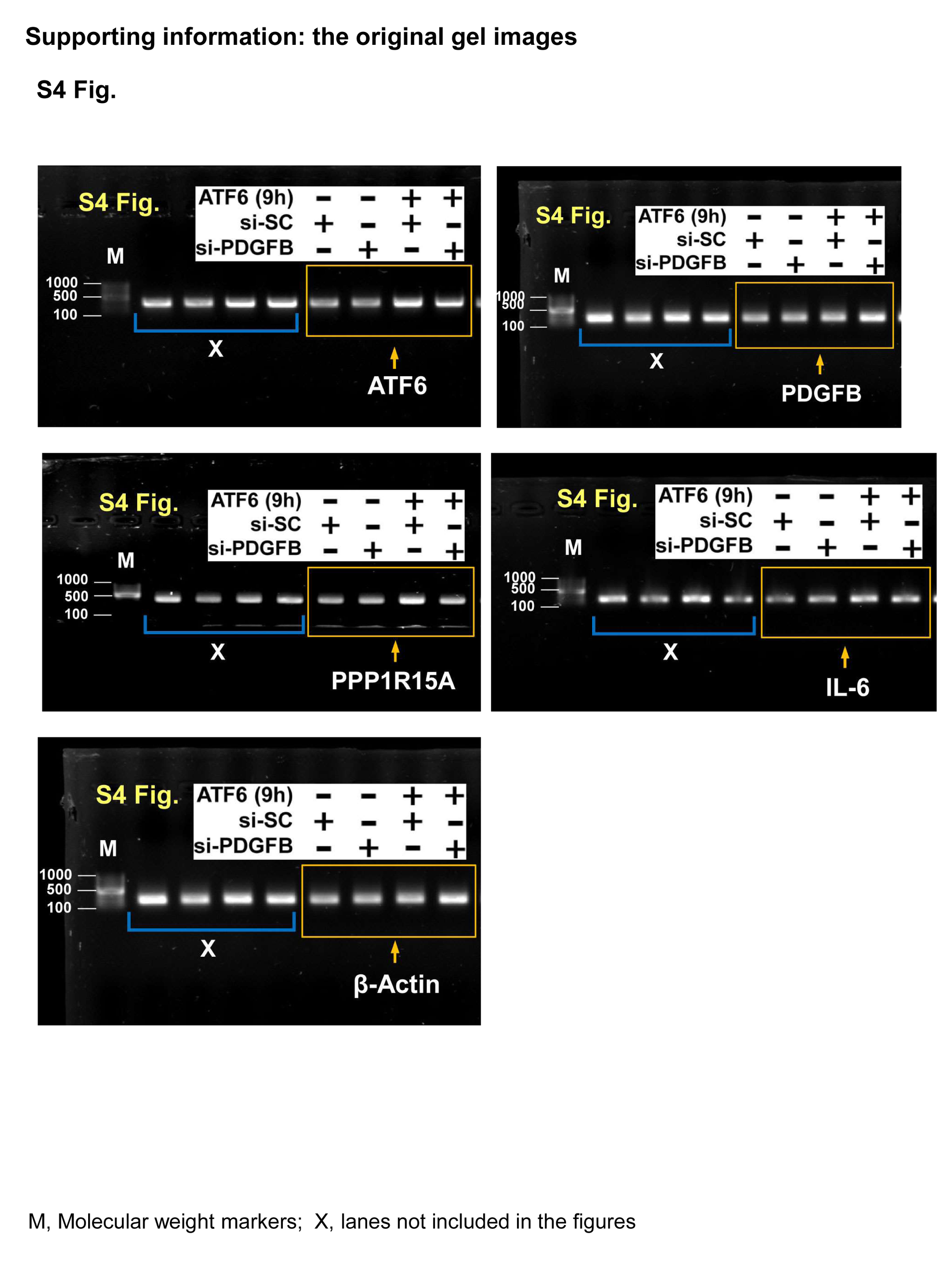

Supplement: S13 Fig — Data represnet the raw uncropped images of S4 Fig. M, Molecular weight markers; X, lanes not included in the figures. (TIF) [file pone.0309749.s013.tif]
