# Supplementary material for: Transcriptomic analysis of cellular senescence induced by ectopic expression of ATF6α in human breast cancer cells
Source: PLoS One. 2024 Oct 28;19(10):e0309749. doi: 10.1371/journal.pone.0309749 (PMC11515977; doi:10.1371/journal.pone.0309749)
Supplement: Attachment — Submitted filename: Response to Reviewers (revision).docx [file pone.0309749.s015.docx]

July 8, 2024

Dr. Aniruddha Datta

Academic editor

PLOS ONE

Dear Editor,

We are submitting our revised manuscript entitled ‘Transcriptomic analysis of cellular senescence induced by ectopic expression of ATF6α in human breast cancer cells’ (PONE-D-24-15574) for publication as research article in PLOS ONE. We have revised the manuscript in accordance with the reviewers’ comments. Attached, please find our responses to the journal’s requirements and the reviewer’s comments. We are going to upload ‘Answers to the Reviewer’s comments’ as a separate file labeled ‘Response to Reviewers’.

**[Answers to the Reviewers’ comments]**

**• Reviewer #1**

1. However, I would personally it more reader-friendly and it would improve the chances of receiving more citations if the authors were add more content specifically around the phenotypic manifestations of the 20 genes they found were significant in their time-series analysis and also find ways to weave a few lines on how their phenotypic manifestations might be inter-related. Such description can also be extended to offer the author’s proposed strategies on how that might be used to provide more intuition around personalizing cancer therapies.

Thank you for the helpful comments. We have added a “Function” column in Table 3 for the 20 DEGs identified in the time-series analysis. For consistency, we have also added a “Function” column to Table 1 and Table 2.

2. Also, I would much prefer the authors shared their NGS sequencing data files.

We have deposited our **RNAseq** data files in the Gene Expression Omnibus (GEO) database. The data generated in this study is available in the GEO database under accession code GSE270286. The URL for accessing the data is <https://www.ncbi.nlm.nih.gov/geo/query/acc.cgi?acc=GSE270286>, and the secure token needed to access the data is ilmjkwmedzyxdsb.

**• Reviewer #2**

1. It requires little more details about the motivation of using 6h and 9h DEGs.

In this study, our focus was on the early-stage transcriptomic changes following the induction of cellular senescence through ectopic expression of ATF6α. We selected the time points of 6 and 9 hours based on our observation that mRNA expression of ATF6α target genes began to increase as early as 6 to 9 hours after induction in MCF-7 cells.

2. It also lacks the details of how specifically MAP2K 1/2-DDIT3 axis influences cellular senescence via ATF6α expression. The paper does not mention how other could have play some roles in linking the axis.

ATF6α overexpression or activation appears to induce MAP2K1/2 (MEK1/2) activation, subsequently enhancing DDIT3 activity to initiate senescence. The mechanism by which ATF6α activates MAP2K1/2 remains unclear. However, MAP2K1/2 function as upstream kinases of ERK1/2, a pathway well-documented in cellular senescence (Lin et al., Genes Dev. 1998 12:3008; Wang *et al.*, Mol. Cell. Biol. 2002 22:3389). The MAP2K-ERK pathway is known to induce cellular senescence, and ERK1/2 has been reported to activate DDIT3 expression (Lawrence et al., Proc. Natl. Acad. Sci. USA 2007 104: 11518; Oh et al., J. Biol. Chem., 2010 285:41310).

**• Reviewer #3**

1. The authors address an important question on the role of ATF6α in cellular senescence. However, the manuscript can made robust by
1) Are the DEGs the same if they work in a different cell line system?

In our previous work, we found that the ectopic expression of ATF6α alone was sufficient to cause cellular senescence in both MCF-7 human breast cancer cells and Caki-1 human renal cancer cells. However, we have not yet performed RNAseq analysis in Caki-1 cells. We expect that the DEGs from Caki-1 cells will overlap with those identified in MCF-7 cells, and that the overlapping DEGs will include crucial genes involved in the induction of cellular senescence. However, considering that the transcriptomic and proteomic patterns of cellular senescence are known to be heterogeneous depending on the cell type and senescence-inducing stimuli, many of the DEGs may differ between the cell lines.

2) The use of ATF6α as a downstream O/E gene for their analysis. This is a little counterintuitive as they are over expressing the gene and hence this is to be expected.

To reveal the DEGs underlying cellular senescence induced by the ATF6 branch of the ER stress signaling pathway, we ectopically expressed ATF6α. Thus, increased expression of ATF6α serves as a positive control.

3) Can they relate this to patient survival data between patients having high and low ATF6α expression levels? That will provide a nice translation link to their work.

Thank you for your valuable comments. We have analyzed the overall survival (OS) of breast cancer patients with high and low expressions of ATF6α using Kaplan-Meier plotter (<https://kmplot.com/analysis>). The analysis shows that breast cancer patients with high ATF6α expression levels have decreased OS rates compared to those with low ATF6α expression levels. The Kaplan-Meier plots were added in the Supporting Information section as S5 Fig. in the revised manuscript. In addition, breast cancer patients with high expression levels of MAP2K1, MAP2K2, or DDIT3—genes on the ATF6α-MAP2K1/2-DDIT3 axis—also showed decreased OS rates compared to patients with low expression levels of these genes (S5 Fig.), supporting the notion that ATF6α, MAP2K1/2, and DDIT3 function in a senescence-inducing signaling axis. These findings are consistent with the idea that cellular senescence contributes to chemoresistance, epithelial-mesenchymal transition (EMT), metastasis, and/or the production of cancer stem cells (CSCs). We also mentioned this point in the revised manuscript (line 378-389).

4) The biological mechanism of ATF6α ectopic expression is lacking and could be outside the scope of this work.

We previously reported that cellular senescence is induced by the abrogation of neighbor of BRCA1 gene 1 (NBR1), UV irradiation, and H-RasV12 overexpression through the ER stress-UPR/ATF6α axis in MCF-7 cells (Kim HS et al., FASEB J., 33(2): 2422-2434, 2019). These findings suggest that cellular senescence is induced through ER stress, with ATF6α playing a crucial role among the three branches of the unfolded protein response (UPR). As an extension of this study, we aimed to gain insight into the downstream molecular targets of ATF6α by performing transcriptomic analyses in MCF-7 cells after ectopically expressing ATF6α.

**• Reviewer #4**

1. Page 5**:** Line 62-64, please cite the study for the finding. If it is part of reference 1, please recite….

As the reviewer suggested the reference is recited after the sentence in lines 62-64.

2. Line 68-70: can the authors provide the rationale behind studying cell senescence in cancer cell lines and the broader impact of it?

Cellular senescence induced during cancer therapy may cause therapy resistance, metastasis and generation of cancer stem cells. Thus, it is important to inhibit senescence during cancer therapy. Our study is a part of an effort to elucidate the mechanism of cellular senescence in cancer cells and to develop a way to reduce it. The rationale behind studying cellular senescence in cancer cell lines were described in the 3^rd^ paragraph of the ’Introduction’ (lines 86-95) in the original submitted manuscript. It is now moved right after the 1^st^ paragraph (lines 59-70) in the revised manuscript.

3. Line 86-93: introduces the importance of cell senescence in this study and should be discussed earlier. Either immediately after line 70 or the authors can briefly discuss why cell senescence is important to study in cancer cell in the paragraph of Line 59-70.

The 3rd paragraph (lines 86-95) has been moved immediately after line 70 of the 1st paragraph.

4. Line 99-100: Why did the authors pick MCF-7 cell line?

We chose MCF-7 as a representative human breast cancer cell line. MCF-7 is one of the most widely used human breast cancer cell lines with an ER(+)/PR(+)/HER2(-) phenotype. This phenotype represents more than 70% of human breast cancers. We have described the point in the Introduction section (line 69-73) and the Discussion section (line 492-493) in the revised manuscript.

5. Line 225: Have any of these 6 genes been implicated in cell senescence before?

The three genes, *ATF6, DDIT3, ATF3*, have been implicated in cell senescence before. However, the other three genes, DNAJB9, CHAC1, HERPUD, are poorly understood in cellular senescence. We have added following sentence in the Discussion section (line 434-438) in our revised manuscript:

“Taken together, among the six common denominator genes, *ATF6, DNAJB9, CHAC1, HERPUD1, DDIT3, and ATF3*, of 6-DEGs and 9-DEGs (Table 1), the ATF6, DDIT3, and ATF3 genes were implicated in cellular senescence. The roles of the DNAJB9, CHAC1, HERPUD1 genes in senescence induction have not been reported but these genes are all involved in tumorigenesis in breast cancers [38-40]”

Thank you for considering our manuscript for publication.

Best Regards

Jeongwon Sohn, M.D., Ph.D.

Professor

Department of Biochemistry and Molecular Biology

Korea University College of Medicine
